# Gastroblastoma: a case report and literature review

**DOI:** 10.3389/fonc.2026.1739216

**Published:** 2026-04-15

**Authors:** Yan Li, Rui Xu, Qi Wu, Yufei Liu, Chenyu Zhu

**Affiliations:** 1 The First College of Clinical Medical Science, China Three Gorges University, Hubei, Yichang, China; 2 Department of Gastrointestinal and Colorectal Surgery, Yichang Central People’s Hospital, Hubei, China; 3 Department of Pathology, Yichang Central People’s Hospital, Yichang, China

**Keywords:** biphasic tumor, case report, gastroblastoma, GLI1 fusion, hedgehog signaling pathway

## Abstract

**Objective:**

Gastroblastoma (GB) is an extremely rare biphasic malignant embryonal tumor of the stomach, primarily seen in children, with limited case reports in adults. Due to overlapping histological features with other gastric tumors, its definitive diagnosis and the establishment of standardized treatment strategies remain challenging.

**Case report:**

This paper reports an 18-year-old adult male patient who presented with abdominal pain. Preoperative imaging studies revealed a 3.8 cm mass in the gastric antrum, with an initial diagnosis of Gastrointestinal Stromal Tumor (GIST). The patient underwent laparoscopic subtotal gastrectomy. Postoperative histopathological examination showed the tumor was composed of epithelioid and spindle cell mesenchymal components, exhibiting biphasic differentiation. Immunohistochemistry (IHC) results showed positivity for Pan-Cytokeratin (PCK), CD56, and Vimentin, with a low Ki-67 index (5%). A final diagnosis of GB (pT4aN2aM0) was confirmed. The patient did not receive adjuvant therapy postoperatively and showed no signs of tumor recurrence or metastasis at 14 months of follow-up.

**Conclusion:**

The diagnosis in this case was established based on the characteristic histological morphology and immunohistochemical features. Although molecular testing has been shown to provide significant auxiliary value in previous studies, the patient did not undergo relevant testing due to personal preferences. Molecular studies indicate that abnormal activation of the Hedgehog/GLI signaling pathway is its core molecular pathological basis, often involving fusion genes such as *MALAT1-GLI1*. Radical surgical resection is the primary treatment modality, and the overall prognosis is favorable. Future efforts require strengthening multi-center collaboration, further investigating the molecular mechanisms, and developing individualized treatment strategies, including those targeting the Hedgehog pathway, to refine diagnostic and therapeutic guidelines and improve patient outcomes.

## Introduction

Gastroblastoma (GB) is an embryonal-like malignant tumor originating from the muscularis propria, characterized by epithelioid and mesenchymal biphasic differentiation. This disease is extremely rare. Since it was first reported by Miettinen et al. in 2009 (1), fewer than 40 cases have been reported globally, occurring mainly in adolescents, with adult cases being even rarer. The rarity of GB has led to slow progress in researching its pathogenesis, clinical diagnosis, and treatment strategies; to date, no unified diagnostic and treatment guidelines have been formed. Histologically, GB is often clinically misdiagnosed due to its non-specific imaging findings and overlapping features with other gastric sarcomas, such as Gastrointestinal Stromal Tumors (GIST). In recent years, molecular pathology research has revealed the key molecular basis of GB: the abnormal activation of the Hedgehog/GLI signaling pathway, often driven by fusion genes like *MALAT1-GLI1*, providing new directions for the diagnosis and potential targeted therapy of this disease. Although radical surgical resection is currently the main and effective treatment, the adjuvant treatment plan for locally advanced or metastatic cases remains controversial. Recent studies indicate that observation is a viable approach for localized GB after R0 resection. We herein present the clinical and pathological findings of a rare case, while summarizing current literature regarding the molecular landscape and treatment modalities of GB. This study aims to enhance diagnostic precision and inform individualized clinical decision-making. To ensure patient confidentiality, all data have been de-identified according to standard privacy protocols.

## Case report

A male patient, 18 years old, presented to our hospital due to “abdominal pain for 2 months”. Two months ago, the patient developed intermittent, nonspecific abdominal pain and mild anorexia. The clinical course was unremarkable for nausea, vomiting, hematochezia, or weight loss. Given the persistence of symptoms initially attributed to a general gastrointestinal disorder, diagnostic imaging was subsequently performed. The patient had been in good health previously, denied any history of chronic underlying diseases such as hypertension or diabetes, no history of infectious diseases like hepatitis B, tuberculosis, or syphilis, and no family history of malignant tumors. The psychosocial background was not further explored. Physical examination revealed a soft abdomen, mild tenderness on palpation in the middle-upper abdomen, no rebound tenderness, and no abnormal masses palpated in the entire abdomen.

**Figure 1:**
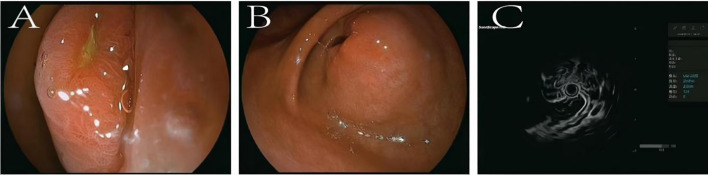
Endoscopic ultrasound and miniprobe ultrasound of the gastric antrum. **(A)** The mucosa exhibits alternating red and white areas, with the red areas predominating, and a 4 cm protrusion is visible. **(B)** A smooth surface with localized depression and ulceration, showing irregularities in the mucosal layer. **(C)** A suspected hypoechoic lesion originating from the muscularis propria, with unclear boundaries due to the limited scanning range of the miniprobe ultrasound.

**Figure 2:**
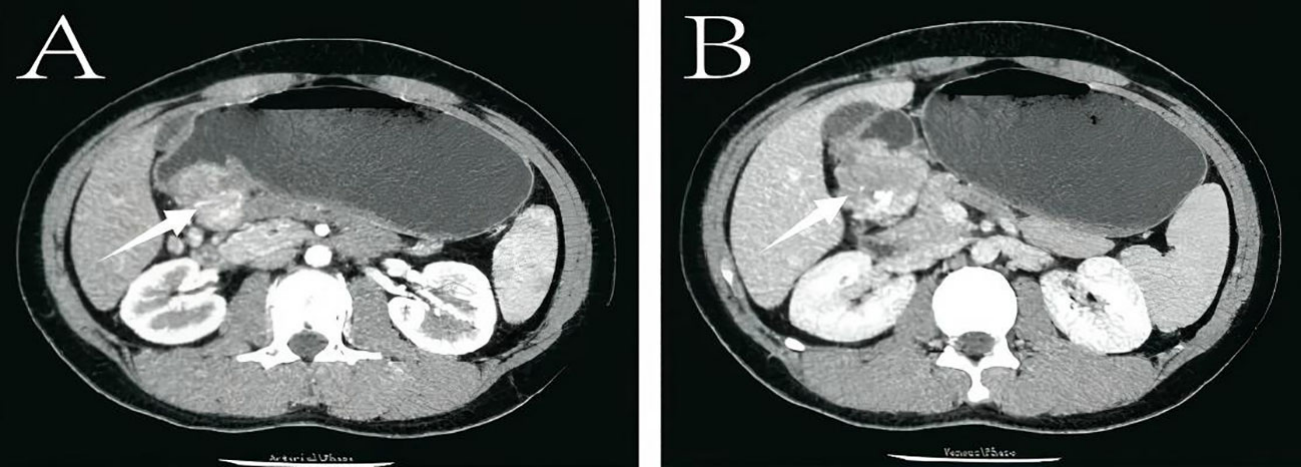
Contrast-enhanced computed tomography (CT) results. **(A)** Arterial phase, showing a mass-like density increase (3.8×3.6 cm) in the gastric antrum with patchy high-density calcifications, relatively well-defined margins, and significant enhancement, accompanied by enlarged surrounding lymph nodes. **(B)** Venous phase, displaying similar characteristics with well-defined boundaries and significant enhancement, along with enlarged lymph nodes.

Relevant examinations were completed after admission. Laboratory tests, including complete blood count, biochemistry (liver and renal function, electrolytes), and tumor markers (CEA, CA19-9), were all within normal limits. All tumor marker results are summarized in **Table 1**. There were no biochemical signs of infection (normal CRP and negative cultures) or other systemic complications. Endoscopic ultrasound (EUS) revealed a submucosal hypoechoic protrusion in the gastric antrum, considering the possibility of a stromal tumor (**Figure 1**). Contrast-enhanced CT showed a mass with calcification in the gastric antrum, measuring approximately 3.8×3.6 cm, with enlarged surrounding lymph nodes, highly suggestive of a GIST originating from the gastric wall (**Figure 2**). Based on imaging and endoscopic findings, the preoperative diagnosis was likely: Gastrointestinal Stromal Tumor.

**Table 1:** Tumor markers.

Indicator	Result	Reference range
Alpha-fetoprotein (AFP)	0.9 ng/L	0~13.6 ng/L
Carcinoembryonic antigen (CEA)	1.5 ng/L	0~6.5 ng/L
Carbohydrate antigen 199 (CA19-9)	3.2 U/mL	0~39 U/mL
Carbohydrate antigen 125 (CA12-5)	0.9 IU/mL	0~6.9 IU/mL

All indicators were within the normal range, and infection markers were not elevated.

**Figure 3:**
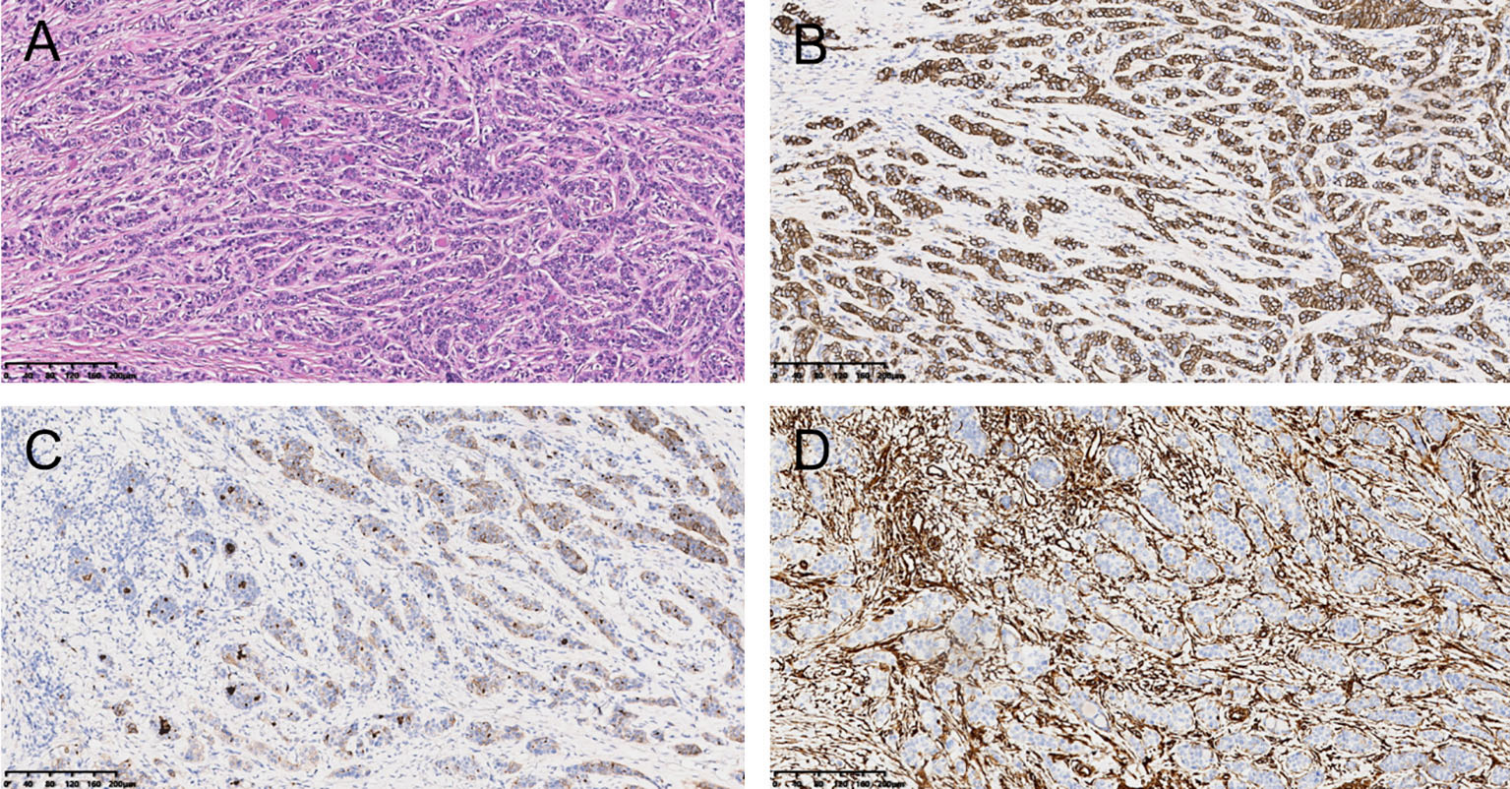
Histopathological and immunohistochemical features of gastric gastroblastoma. **(A)** Hematoxylin and eosin staining demonstrates the characteristic biphasic architecture composed of epithelial structures and spindle cell components. **(B)** CD56 immunohistochemistry shows diffuse positivity in tumor cells. **(C)** CD10 immunostaining reveals focal positivity in tumor cells. **(D)** Vimentin immunostaining highlights strong expression in the spindle cell component. (All images at ×100 magnification; scale bar = 200 μm.

Following a multidisciplinary preoperative assessment, a laparoscopic subtotal gastrectomy with gastrojejunostomy and regional lymphadenectomy was planned. This approach was selected based on: (1) the distal localization of the tumor in the gastric antrum and the absence of surgical contraindications; (2) the minimally invasive nature of laparoscopy, which facilitates accelerated postoperative recovery; and (3) the necessity of comprehensive lymph node staging for guiding subsequent therapeutic strategies. After thorough informed consent regarding potential risks, the patient underwent the procedure on July 5, 2024. Intraoperative exploration revealed a firm, 5.0×5.0 cm mass in the gastric antrum. No evidence of serosal invasion, adjacent organ involvement, or peritoneal seeding was observed. An oncologically sound resection was performed to ensure negative margins (R0 resection). Postoperative histopathological examination was key to the definitive diagnosis. The gross specimen showed a grayish-white mass with a maximum diameter of 4.2cm, solid on cross-section. The tumor invaded the full thickness of the gastric wall, locally reaching the serosa; cancer emboli were visible in the vessels; no perineural invasion was seen; both surgical margins and the omentum were free of tumor. Tumor (epithelial component) metastasis was seen in the perigastric lymph nodes (4/17). Microscopic examination showed biphasic differentiation of the tumor (**Figure 3A**): the majority of tumor cells were epithelioid, arranged in nests, cords, and glandular patterns; the cytoplasm was partially pale-staining, nuclei were round, nucleoli were inconspicuous, and mitotic figures were rare; a small portion of tumor cells were spindle-shaped; localized calcification was visible. Immunohistochemistry: Epithelioid cells expressed CD56 (**Figure 3C**) and PCK, partially expressed CD10 (**Figure 3D**) and TFE3; Spindle cells expressed CD56 (**Figure 3C**), CD10 (**Figure 3D**), and Vimentin (**Figure 3B**), partially expressed TFE3; CgA, Syn, S-100, CD99, INSM1, HMB-45, CR (Calretinin), WT-1, and SS18-SSX were negative in both cell types. The Ki-67 labeling index was about 5%. Combined with clinical data, microscopic findings, and IHC results, the pathological diagnosis supported GB. The pathological stage was pT4aN2aM0.

**Figure 4:**
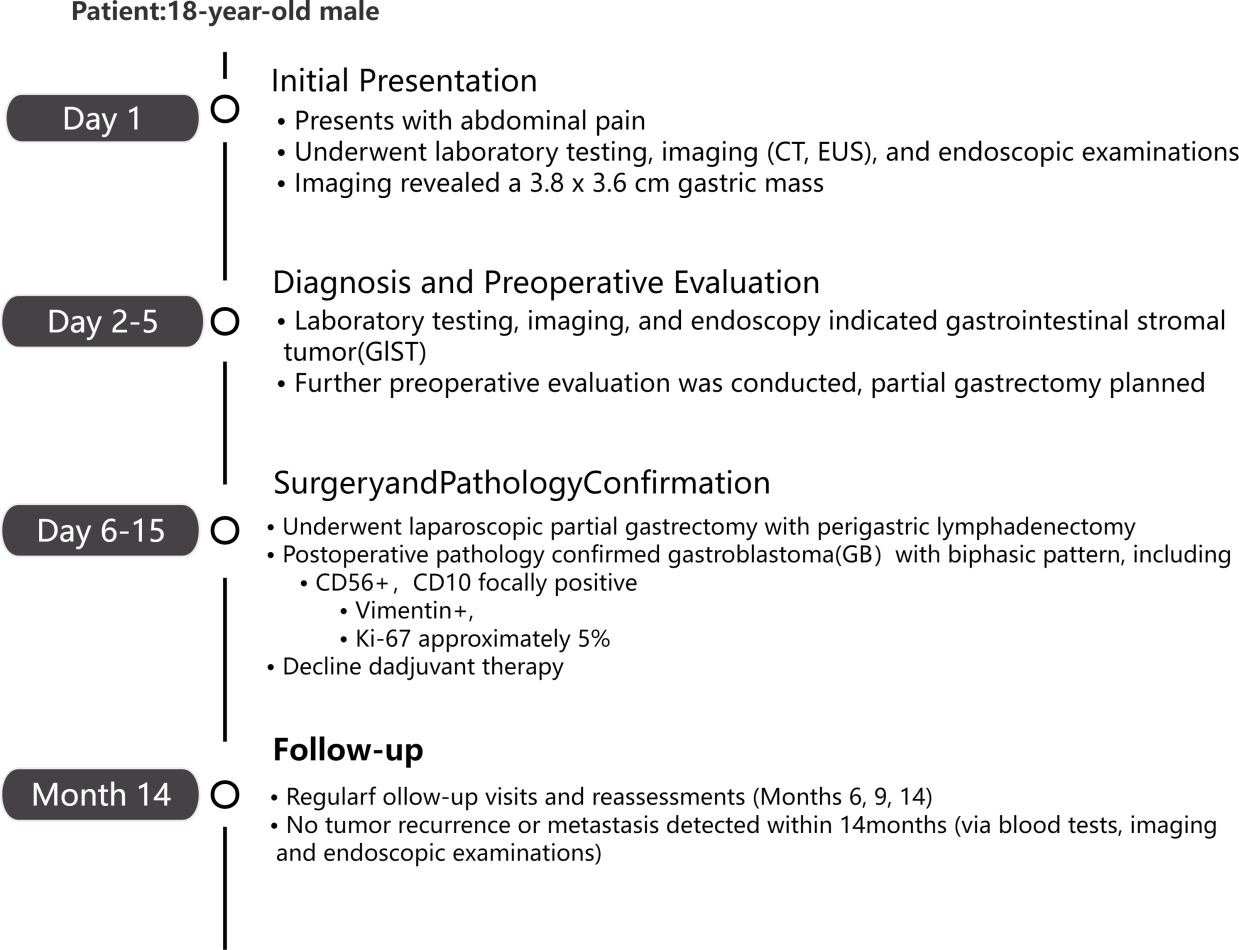
Timeline of diagnosis and treatment for an 18-year-old male with gastroblastoma.

Although postoperative adjuvant therapy was highly recommended given the advanced pathological stage, the patient declined further treatment following comprehensive counseling regarding the associated risks. The patient opted for observation and postoperative convalescence. At the 14-month follow-up, radiographic evaluations demonstrated no evidence of disease (NED), with no signs of local recurrence or distant metastasis. The patient remains in good clinical condition and will continue under close active surveillance.The detailed diagnostic and therapeutic workflow is shown in **Figure 4**.

The patient recovered well postoperatively and met the criteria for discharge on July 16, 2024. The patient did not receive adjuvant chemotherapy or radiotherapy. As of the writing of this report, the patient has been followed up for 14 months, is in good general condition, and shows no local tumor recurrence or metastasis on imaging review and clinical assessment; the patient’s condition continues to be monitored dynamically.

## Discussion

Gastroblastoma (GB) is a highly malignant gastric tumor with unique epithelio-mesenchymal biphasic differentiation. It predominantly affects adolescents or young adults, and its rarity results in insufficient clinical experience regarding its diagnosis and treatment. Herein, we characterize the clinical, histological, and immunophenotypic hallmarks of a rare GB in a young adult. Furthermore, we provide a comprehensive 15-year literature review elucidating its molecular alterations. This report aims to offer updated, evidence-based insights to inform the precise diagnosis and clinical management of this rare malignancy.

The clinical and imaging features of GB lack specificity, posing a significant challenge to preoperative diagnosis. Based on a comprehensive review of domestic and international literature, a cumulative total of 32 GB cases (including the present case) have been reported to date, with 19 cases reported abroad and 13 domestically (2–4). The clinicopathological features, molecular alterations, treatment regimens, and prognosis of all cases are detailed in **Table 2**. Statistical analysis shows that GB primarily affects young patients, with a wide age range at onset (5 to 74 years), and a similar incidence in males and females, showing no significant gender predilection. Anatomically, GB most commonly involves the gastric antrum (20/32 cases, 62.5%), followed by the gastric body (8/32 cases, 25.0%) (8, 10, 11). Clinical manifestations are often non-specific, such as abdominal pain, melena, and fatigue; a few patients are discovered incidentally during physical examination. Radiologically, GB often presents as a relatively well-demarcated submucosal solid mass, making it easily confused with other mesenchymal-derived tumors, such as GIST or glomus tumors, in the differential diagnosis. The experience from our case corroborates this: the patient’s preoperative imaging and endoscopic assessments both favored GIST. However, after intraoperative exploration confirmed a localized lesion and successful radical (R0) resection was performed, the definitive diagnosis was ultimately dependent on postoperative histopathology and immunohistochemistry results. This case further underscores the indispensable and critical role of pathological and immunohistochemical analysis in establishing the diagnosis of GB (12, 14).

**Table 2:** Clinicopathological characteristics of gastroblastoma.

Case no.	Reference	Age (years)/sex	Location	Clinical features	Treatment	Immunohistochemistry (IHC)	Fusion gene	Exon breakpoint	Metastases/relapse	Outcome	Follow up (month)
1	Miettinen et al. (1)	19/M	Greater curvature of the stomach	Nonspecific abdominal pain	SG	EC:AE1/AE3, CK18, CK7 SC:Vimentin, CD10	Not performed	—	NO	ANED	42
2	Miettinen et al. (1)	27/F	Greater curvature of the stomach	Nonspecific abdominal pain	PG	EC:AE1/AE3, CK18, CK7 SC:Vimentin, CD10	Not performed	—	NO	ANED	60
3	Miettinen et al. (1)	30/M	Gastric antrum	Anemia, fatigue	PG+RT	EC:AE1/AE3, CK18, CK7 SC:Vimentin, CD10	Not performed	—	NO	ANED	168
4	Shin et al. (2)	9/M	Gastric antrum	Abdominal pain, periumbilical mass	PG	EC:CD56, AE1/AE3, LMWCK, EMA, CD117 SC:CD56, Vimentin, CD10	*MALAT1-GLI1*	*MALAT1exon 1– GLI1 exon 6*	NO	ANED	93
5	Wey et al. (3)	28/M	Gastric antrum	Constipation	CT+PG	EC:CD10, CD56, CK, CK7, CD117 SC:CD10, CD56, Vimentin	*MALAT1-GLI1*	*MALAT1exon 1– GLI1 exon 6*	Liver, lymph node, retroperitoneal and bladder	AWD	3
6	Fernandes et al. (4)	19/F	Gastric antrum	Abdominal pain	PG	EC:CD56, CD10, AE1/AE3, CAM5.2 SC:CD56, CD10, Vimentin	Not performed	—	NO	ANED	20
7	Ma et al. (5)	12/M	Gastric antrum	Intermittent blood in stool, abdominal mass	SG	EC:AE1/AE3, CAM5.2 SC:Vimentin, CD10, CD56	Not performed	—	NO	ANED	8
8	Toumi et al. (6)	29/F	Gastric body	Epigastric pain	PG+ST	EC:CD10, CD99, Vimentin SC:CD10, CD99, Vimentin	Not performed	—	Splenic, Lymph node/Local relapse	D	7
9	Graham et al. (7)	27/M	Gastric antrum	NA	R	EC:pan-CK SC:SMA	*MALAT1-GLI1*	MALAT1exon 1– GLI1 exon 6	NO	ANED	12
10	Graham et al. (7)	56/F	Stomach	NA	EGD	EC:CK SC:Vimentin	*MALAT1-GLI1*	*MALAT1exon 1– GLI1 exon 6*	Liver	NA	NA
11	Zhu et al. (8)	65/F	Greater curvature of the stomach	Physical examination	EFTR	EC:CD56, CD10, CD99, NSE, pan-CK, Vimentin SC:CD56, CD10, CD99, NSE	Not performed	—	NO	ANED	3
12	Castri et al. (9)	74/M	Gastric antrum	Weight loss and dysphagia	PG	EC:CD56, CD10, Bcl2, AE1/AE3 SC:CD56, CD10, Bcl2, Vimentin	*MALAT1-GLI1*	*GLI1 exon 6* (retained)	Local relapse	AWD	52
13	Centonze et al. (10)	43/F	Gastric antrum	Intestinal bleeding	PG	EC:GLI1, pan-CK, LMWCK, EMA SC:GLI1, Vimentin, CD10	Not performed	—	NO	ANED	100
14	Long et al. (11)	53/F	Greater curvature of the stomach	Abdominal pain	R	EC:CD10, CD56, Vimentin, pan-CK, EMA SC:CD10, CD56, Vimentin	Not performed	—	NO	ANED	14
15	Koo et al. (12)	17/M	Gastric fundus	Hematemesis and melena	PG	EC:Vimentin, CD56, CD10, pan-CK, Syn SC:Vimentin, CD56, CD10, pan-CK, Syn	*EWSR1-CTBP1*	Not reported	NO	ANED	23
16	Chen et al. (13)	58/M	Lesser curvature of the stomach	Physical examination	ESD	EC:Vimentin, CD10, bcl-2, CD56, S100, EMA SC:Vimentin, CD10, bcl-2	*PTCH1-GLI2*	*PTCH1 exon 1 - GLI2 exon 8*	NA	NA	NA
17	Chen et al. (14)	43/M	Gastric antrum	Regurgitation and melena	PG	EC:pan-CK SC:Vimentin, CD10, CD56	Not performed	—	NO	ANED	24
18	Li et al. (15)	55/M	Gastric angle	Physical examination	ESD	EC:CD56, pan-CK, EMA SC:CD56, Vimentin, CD10	*ACTB-GLI1*	*ACTB exon 1 - GLI1 exon 3*	NO	ANED	12
19	Sugimoto et al. (16)	28/F	Gastric antrum	Abdominal pain	PG	EC:pan-CK, CAM5.2, CK7 SC:Vimentin, CD10, CD56	*MALAT-GLI1*	Not reported	NO	ANED	8
20	Gong et al. (17)	19/F	Gastric antrum	Loss of appetite and lost body weight	PG	EC:CD10, CD56, EMA, pan-CK SC:CD10, CD56, Vimentin	Not performed	—	NO	ANED	19
21	McCammon et al. (18)	26/M	Gastric pylorus	Abdominal pain	R	EC:CD56, CD10, AE1/AE3 SC:CD56, CD10	*MALAT1-GLI1*	Not reported	NO	ANED	2
22	Feng et al. (19)	5/F	Greater curvature of the stomach	Abdominal pain and melena	PG	EC:Vimentin, Bcl2, GLI1, CD56, CAM5.2, CD10, CK SC:Vimentin, Bcl2, GLI1	Not performed	—	NO	ANED	24
23	Li et al. (20)	51/F	Gastric antrum	Melena	PG	EC:CD56, CK, EMA, CKL, CAM5.2 SC:CD56, Vimentin	MALAT1-GLI1	Not reported	NO	ANED	24
24	Pinto et al. (21)	53/F	Gastric antrum	Heartburn and dyspepsia	PG	EC:CD10, CD56, CAM5.2, AE1/AE3 SC:CD10, CD56, Vimentin	Not performed	—	NO	ANED	18
25	Bongrain et al. (22)	28/M	Gastric antrum& Gastric pylorus	Abdominal pain	AT	EC:pan-CK, CD56, CD10 SC:pan-CK, CD56, CD10	*ACTB-GLI1*	Not reported	NO	ANED	50
26	Huang et al. (23)	39/M	Gastric antrum	Regurgitation	ESD	EC:CD56, CD10, SMA, pan-CK SC:CD56, CD10, SMA, pan-CK	*ACTB-GLI1*	Not reported	NO	ANED	12
27	Shabbir et al. (24)	19/M	Gastric antrum	Abdominal pain and loss of appetite	AT	EC:CD56, Vimentin, pan-CK, AE1/AE3 SC:CD56, Vimentin, CD10	*ACTB-GLI1*	Not reported	NO	ANED	9
28	Shibayama et al. (25)	25/F	Gastric body	Hematemesis and anemia	ESD	EC:CD10, SMA, GLI1, SOX2, cyclin D1 SC:CD10, SMA, GLI1, SOX2, cyclin D1	*PTCH1-GLI2*	*PTCH1 exon 1 - GLI2 exon 8*	NO	ANED	60
29	Jin et al. (26)	61/F	Greater curvature of the stomach	Abdominal distension	SG	EC:Vimentin, CK, CD56, CD10, SMA SC:Vimentin, CK, CD56, CD10, SMA	*GLI1* translocation(+)	Not reported	NO	ANED	12
30	Hong et al. (27)	58/M	Lesser curvature of the stomach	Physical examination	ESD	EC:Vimentin, CD10, Bcl2, CD56, EMA, S100 SC:Vimentin, CD10, Bcl2	*PTCH-GLI2*	Not reported	NO	ANED	36
31	Hong et al. (27)	21/M	Gastric antrum	Abdominal pain	R	EC:CD56, CD10, AE1/AE3 SC:CD56, CD10, SMA, CD117, DOG1	*MALAT1-GLI1*	Not reported	NO	ANED	8
32	Present case	18/M	Gastric antrum	Abdominal pain	SG	EC:CD56, PCK, CD10, TFE3 SC:CD56, Vimentin, CD10, TFE3	Not performed	—	NO	ANED	14

ANED, Alive with no evidence of disease; AT, Antrectomy; AWD, Alive with disease; CT, Chemotherapy; D, death; EC, Epithelioid cell; EFTR, Endoscopic full-thickness resection; EGD, Esophagogastroduodenoscopy; ESD, Endoscopic submucosal dissection; F, Female; M, Male; LR, Local recurrence; PG, Partial gastrectomy; R, Resection; RT, Radiotherapy; SC, Spindled cell; SG, Subtotal gastrectomy; ST, Splenectomy; NA, Not available.

The key to diagnosing GB lies in its unique histopathological features and immunophenotype. Histologically, GB presents as a nodular or lobulated solid mass, often with focal cystic changes, hemorrhage, or ulceration. The cut surface is grayish-white to grayish-tan, and the borders are relatively distinct. Microscopically, the tumor is composed of varying proportions of epithelioid cells and mesenchymal spindle cells, forming the diagnostic Biphasic Pattern. Immunohistochemical results further confirm this biphasic differentiation: the epithelial component shows strong positivity for Pan-CK, CAM5.2, EMA, and CD56, with partial expression of CK7, CK8, and CD10; the stromal component expresses Vimentin, CD10, and CD56. Concurrently, negative results are crucial for differential diagnosis, as markers such as DOG1, CD117, CgA, Calretinin, SMA, and SS18-SSX are typically not expressed (16).

In recent years, breakthroughs in molecular pathology have significantly deepened our understanding of GB pathogenesis (17). Studies indicate that abnormal activation of the Hedgehog/GLI signaling pathway plays a critical role. In the canonical Hedgehog (HH) signaling pathway, the transmembrane receptor *PTCH1* constitutively represses SMO in the absence of ligands. Upon ligand (e.g., Sonic Hedgehog, SHH) binding to *PTCH1*, SMO is de-repressed, prompting the dissociation of SUFU from the *GLI* complex. This prevents the cleavage of *GLI2/3* into repressor forms, allowing full-length, activated *GLI* proteins to translocate to the nucleus. As the terminal transcriptional effector, *GLI1* amplifies this cascade by inducing target genes—such as *PTCH1* (negative feedback), CCND1 (cell cycle progression), SOX2 (stemness), and VEGFA (angiogenesis)—thereby driving cellular proliferation and preserving an undifferentiated phenotype (28, 29).

In 2017, Graham et al. first identified the *MALAT1-GLI1* fusion in GB (1). The resulting chimeric *GLI1* protein retains its zinc-finger DNA-binding and transcriptional activation domains, driving persistent, ligand-independent overexpression of *GLI1*. This bypasses upstream regulation, culminating in the constitutive activation of the HH pathway, which mechanistically underpins the ‘embryonal’ histomorphology and high proliferative index characteristic of GB. Notably, *GLI* acts as a signaling hub that can also be modulated via non-canonical crosstalk with pathways such as PI3K/AKT, RAS/MAPK, and TGF-β (30). The emergence of other rare fusions in GB, including *PTCH1-GLI2*, *ACTB-GLI1*, and *EWSR1-CTBP1*, further underscores this complexity (12, 13). Although the precise phenotypic variations driven by these distinct fusions warrant further investigation, aberrant HH pathway activation—primarily orchestrated by *GLI*-related fusions like *MALAT1-GLI1*—is unequivocally the core molecular driver of GB, providing a robust rationale for future precision diagnostics and targeted therapeutics.

Of note, molecular testing for fusion genes was omitted in this case, as the patient declined further genetic analysis following comprehensive counseling. Nevertheless, the diagnosis of GB was unequivocally established based on its hallmark biphasic epithelial and mesenchymal histomorphology, coupled with a congruent immunophenotype. In current clinical practice, GB diagnosis relies predominantly on these morphologic and immunohistochemical parameters, rendering molecular analysis a valuable adjunct rather than an absolute prerequisite. Nonetheless, identifying *GLI*-related fusions can substantiate the underlying oncogenic drivers and potentially inform future targeted therapeutic strategies. Therefore, we advocate for the incorporation of molecular profiling in the diagnostic workup of GB whenever clinically feasible and aligned with patient preference.

Importantly, *GLI*-driven fusions are not exclusive to GB, necessitating careful morphologic and immunophenotypic distinction from other entities. For instance, GISTs share the gastric location but exhibit a monophasic spindle cell morphology and distinct CD117/DOG1 positivity (1). Similarly, other *MALAT1-GLI1*-positive fibromyxoid tumors display monomorphic spindle cells and lack the cytokeratin expression defining GB’s epithelial component (31). Moreover, *ACTB-GLI1*-rearranged perivascular tumors typically occur in head and neck soft tissues and feature a concentric cellular arrangement around vessels (32), while *EWSR1*-associated *GLI1*-rearranged tumors often present an undifferentiated small round-cell phenotype (33). Therefore, despite a shared underlying oncogenic driver, the final diagnosis of GB relies predominantly on its anatomical site, classic biphasic morphology, and supportive immunophenotype, positioning molecular testing as a complementary diagnostic tool.

Currently, there is a lack of unified, standardized treatment guidelines for GB. Our systematic literature review (see **Table 2**) confirms that surgical resection remains the primary treatment modality, and most patients who undergo radical resection achieve long-term recurrence-free survival (23, 24). For small, early-stage lesions confined to the gastric wall, Endoscopic Submucosal Dissection (ESD) is also considered a minimally invasive alternative with rapid recovery and has been successfully applied in a few previously reported cases (e.g., cases 16, 18). However, for advanced cases with incomplete resection, local recurrence, or distant metastases, surgery alone is insufficient for long-term control. These outcomes suggest that for patients with high-risk factors for recurrence or unresectable lesions, individualized adjuvant or neoadjuvant chemotherapy should be considered, or they should be enrolled in clinical trials.

The experience from our case further supports the primacy of surgery. Based on the tumor’s clinicopathological features and the patient’s performance status, a curative R0 resection was successfully performed, followed by an uneventful postoperative recovery. Given the rarity of GB and the theoretical risk of recurrence, adjuvant therapy was recommended. However, following comprehensive counseling, the patient opted for active surveillance. Notably, the patient remains free of disease at the current follow-up, suggesting that complete surgical excision alone may be sufficient for long-term disease control in localized GB. This clinical observation aligns with previous reports by Miettinen et al. (1) and Graham et al. (7), which demonstrate a relatively low recurrence rate following curative resection, even in patients with high-risk features.This result indicates that for localized GB, complete radical resection is key to achieving long-term survival. Given the rarity of GB and the lack of clinical guidelines, future research urgently needs to focus on developing standardized diagnostic and treatment pathways. More importantly, discoveries based on molecular mechanisms provide new directions for targeted therapy: given that abnormal activation of the Hedgehog pathway is the core molecular pathological basis of GB, Hedgehog pathway inhibitors (such as vismodegib or sonidegib) have potential applications. Furthermore, some studies have detected the expression of PD-L1 and HDAC2 in the tumor stromal components, suggesting that immune checkpoint inhibitors and epigenetic drugs may become effective strategies for future exploration.

The primary strengths of this study include the comprehensive clinicopathological and immunohistochemical characterization of this rare neoplasm, coupled with a systematic integration of current literature on the molecular mechanisms and therapeutic landscape of GB. Nevertheless, notable limitations exist. These include the restricted generalizability inherent to single-case reports and the lack of molecular confirmation for *GLI* fusions due to the patient opting against further testing. Furthermore, the limited follow-up period and the absence of adjuvant therapy preclude definitive conclusions regarding long-term oncological outcomes and the efficacy of systemic treatments.

In summary, the diagnostic and therapeutic experience of this case of GB highlights its complexity and challenges. Given the rarity of GB and its non-specific clinical, endoscopic, and imaging findings, the risk of preoperative misdiagnosis is extremely high. We emphasize that histomorphological and immunophenotypic analyses remain paramount for the definitive diagnosis of GB. Although the literature strongly supports the diagnostic utility of detecting *GLI*-related fusion genes (e.g., *MALAT1-GLI1*), molecular profiling was omitted in this case due to patient preference. Such fusion events activate the Hedgehog signaling pathway, forming the core molecular pathological basis for the “embryonal-like” structure and high proliferative potential of GB tumors. Based on current statistical data, complete radical surgery remains the core and preferred treatment modality for localized GB. For patients with advanced-stage, incompletely resected, or molecularly high-risk disease, combination targeted therapies (e.g., Hedgehog pathway inhibitors) and immunotherapy show potential clinical value. However, their efficacy and safety still require strong evidence-based medical support from future high-quality randomized controlled trials. In conclusion, given the low incidence of GB, there is an urgent need for multi-center collaboration and international data sharing to establish standardized diagnostic and individualized treatment protocols.

## Conclusion

Gastroblastoma (GB) is an extremely rare gastric malignant tumor with epithelio-mesenchymal biphasic differentiation. Due to its highly non-specific imaging and endoscopic manifestations, as well as the inaccessibility of molecular testing, the preoperative diagnosis of GB is extremely challenging. Histopathology and immunohistochemistry are currently the indispensable and key methods for the definitive diagnosis of GB. Molecular pathology studies have confirmed that *GLI*-related fusion genes and the abnormal activation of the Hedgehog signaling pathway they mediate are the core pathogenetic mechanisms of GB. Regarding treatment strategies, we believe that complete radical surgical resection is the primary and preferred means to achieve long-term survival. Despite presenting with a high-risk pathological stage and clear indications for adjuvant chemotherapy, the patient declined systemic treatment in favor of active surveillance. Notably, the patient remained completely free of recurrence or metastasis at the 14-month follow-up. This favorable outcome suggests that following complete surgical clearance (R0 resection), active surveillance may represent a reasonable management strategy for localized GB in the absence of additional adverse prognostic factors. Ultimately, this case provides valuable clinical evidence supporting observation as a viable postoperative paradigm for this rare malignancy. This case report and systematic literature review provide a valuable supplement to the GB case database, deepening the understanding of the clinicopathological features and molecular basis of this rare tumor. Looking ahead, there is an urgent need for international multi-center collaboration and large-sample clinical studies to refine the diagnostic criteria for GB, clarify prognostic factors, and ultimately establish standardized and individualized treatment protocols based on molecular subtyping, thereby significantly improving the level of diagnosis and treatment and the survival prognosis for patients.Notably, as molecular profiling was omitted in the present case, our discussion regarding the underlying oncogenic mechanisms is extrapolated from existing literature rather than derived from direct molecular confirmation.

## Data Availability

The original contributions presented in the study are included in the article/supplementary material. Further inquiries can be directed to the corresponding author.
